# Notoginseng Leaf Triterpenes Ameliorates OGD/R-Induced Neuronal Injury via SIRT1/2/3-Foxo3a-MnSOD/PGC-1*α* Signaling Pathways Mediated by the NAMPT-NAD Pathway

**DOI:** 10.1155/2020/7308386

**Published:** 2020-10-23

**Authors:** Weijie Xie, Ting Zhu, Ping Zhou, Huibo Xu, Xiangbao Meng, Tao Ding, Fengwei Nan, Guibo Sun, Xiaobo Sun

**Affiliations:** ^1^Institute of Medicinal Plant Development, Peking Union Medical College and Chinese Academy of Medical Sciences, Beijing 100193, China; ^2^Beijing Key Laboratory of Innovative Drug Discovery of Traditional Chinese Medicine (Natural Medicine) and Translational Medicine, Beijing 100193, China; ^3^Key Laboratory of Bioactive Substances and Resources Utilization of Chinese Herbal Medicine, Ministry of Education, Beijing 100193, China; ^4^Key Laboratory of Efficacy Evaluation of Chinese Medicine against Glycolipid Metabolic Disorders, State Administration of Traditional Chinese Medicine, Beijing 100193, China; ^5^Key Laboratory of New Drug Discovery Based on Classic Chinese Medicine Prescription, Beijing 100193, China; ^6^Jilin Academy of Chinese Medicine, Changchun 130012, China

## Abstract

**Background:**

Cerebral ischemic stroke (CIS) is a common cerebrovascular disease whose main risks include necrosis, apoptosis, and cerebral infarction. But few therapeutic advances and prominent drugs seem to be of value for ischemic stroke in the clinic yet. In the previous study, notoginseng leaf triterpenes (PNGL) from *Panax* notoginseng stem and leaf have been confirmed to have neuroprotective effects against mitochondrial damages caused by cerebral ischemia *in vivo*. However, the potential mechanisms of mitochondrial protection have not been fully elaborated yet.

**Methods:**

The oxygen and glucose deprivation and reperfusion (OGD/R)-induced SH-SY5Y cells were adopted to explore the neuroprotective effects and the potential mechanisms of PNGL *in vitro*. Cellular cytotoxicity was measured by MTT, viable mitochondrial staining, and antioxidant marker detection *in vitro*.Mitochondrial functions were analyzed by ATP content measurement, MMP determination, ROS, NAD, and NADH kit *in vitro*. And the inhibitor FK866 was adopted to verify the regulation of PNGL on the target NAMPT and its key downstream.

**Results:**

In OGD/R models, treatment with PNGL strikingly alleviated ischemia injury, obviously preserved redox balance and excessive oxidative stress, inhibited mitochondrial damage, markedly alleviated energy metabolism dysfunction, improved neuronal mitochondrial functions, obviously reduced neuronal loss and apoptosis *in vitro*, and thus notedly raised neuronal survival under ischemia and hypoxia. Meanwhile, PNGL markedly increased the expression of nicotinamide phosphoribosyltransferase (NAMPT) in the ischemic regions and OGD/R-induced SH-SY5Y cells and regulated the downstream SIRT1/2-Foxo3a and SIRT1/3-MnSOD/PGC-1*α* pathways. And FK866 further verified that the protective effects of PNGL might be mediated by the NAMPT *in vitro*.

**Conclusions:**

The mitochondrial protective effects of PNGL are, at least partly, mediated via the NAMPT-NAD+ and its downstream SIRT1/2/3-Foxo3a-MnSOD/PGC-1*α* signaling pathways. PNGL, as a new drug candidate, has a pivotal role in mitochondrial homeostasis and energy metabolism therapy via NAMPT against OGD-induced SH-SY5Y cell injury.

## 1. Introduction

Cerebral ischemic stroke (CIS) is one of the leading causes of death worldwide; it has the characteristics of high morbidity, lethality, disability, and recurrence rate [1–3]. The related researches and reports have revealed that cerebral ischemia and reperfusion (I/R) injury (CIRI) is involved in energy metabolism disorders [4], oxidative stress [5], Ca^2+^ overload, excitatory neurotransmitters, apoptosis, and necrosis [5]. Currently, tissue plasminogen activator is the effective pharmacological therapy approved by the Food and Drug Administration for acute ischemic stroke since 1996. Still, its use remains limited due to the narrow therapeutic window [2, 6]. Moreover, some neuroprotective drugs have been developed for treating I/R injury, such as butylphthalide and edaravone [6, 7]. But it is still difficult to meet the needs for clinical treatment of ischemic stroke. Therefore, it is imperative to develop novel therapeutic strategies for stroke.

Recently, nicotinamide adenine dinucleotide (NAD+, NAD), as a simple metabolic cofactor, can regulate metabolism and cell stress responses, including the sirtuin family of protein deacetylases [8–10]. The regulation of NAD+ alterations, decline, and homeostasis can be found in virtually all age-related diseases, including neurodegeneration, stroke, diabetes, and cancer [8–10].

In the early stage of ischemia, a critical reduction of regional cerebral blood flow results in the severe oxygen and glucose deprivation [11, 12], insufficient NAD+ synthesis, and a decrease in the ratio of NAD^+^/NADH. Then, it directly impairs H+ transmission in the oxidative respiratory chain, decreases intracellular ATP synthesis, and thus causes mitochondrial damages and energy metabolism disorders within minutes after ischemia [11]. Compared to other brain cells, neurons have higher energy demand, but their energy reserves are limited [11–13]. Depletion of ATP often triggers the ischemic cascades such as membrane ion pump failure, efflux of cellular potassium, an influx of sodium, chloride, and membrane depolarization [11–13]. These mitochondrial disorders may trigger mitochondrial quality control, recover mitochondrial morphology [14, 15], and further aggravate the multiple pathological progresses of cerebral I/R injury, including excitotoxicity, mitochondrial response, free radical release, acidotoxity, protein misfolding, and inflammation [3–5, 16]. Thus, mitochondrial metabolic disorder of energy is seen as one of the hallmarks of I/R-induced neuronal death. Currently, maintenance of mitochondrial homeostasis is being pursued as a new therapeutic target for stroke treatment and provides valuable insights for clinical strategies [11, 13, 17].

NAMPT is the rate-limiting enzyme for biosynthesizing NAD in mammals. Currently, much evidence supports NAMPT and the NAMPT-NAD+ biosynthesis pathway as therapeutic targets against ischemic stroke, including neuroprotection, vascular repair, and neurogenesis [8, 10, 18]. Base on the current reports, NAMPT could increase neuronal ischemic tolerance, inhibit neuronal apoptosis necrosis, and improve mitochondrial energy metabolism under ischemia [19–21]. On the one hand, NAMPT strongly reduces MMP depolarization, suppresses ischemia-induced neuronal death via inhibiting the activation of mitochondrial apoptotic signaling pathways, and promotes neuronal survival through inducing autophagy via regulating the mTOR–S6K1 signaling pathway [22, 23]. NAMPT is critically involved in the regulation of such forms of cell death as apoptosis [20, 24] and necroptosis [23, 25] via connecting to sirtuin (SIRT) signaling [18, 23], which constitutes a robust endogenous defense system against various stresses. On the other hand, NAMPT plays a dual role in redox metabolism and biological signaling via the NAMPT-NAD+ biosynthesis pathway [8, 10], which is tightly associated with mitochondrial functions [19, 20, 24], AMPK signaling activation [26–28], and SIRT deacetylase activity alterations under ischemia stress [18, 26]. All of these reports suggest that NAMPT is a crucial target for the prevention and treatment of CIS. Therefore, it is one of the hot tasks to find natural active substances and compounds around NAMPT targets for the prevention and treatment of ischemic stroke.

Ischemic stroke is a complex pathological process with multiple mechanisms [1–3]. Many pharmacological agents have been investigated for years, though with limited clinical success. Thus, it is appropriate to consider using pharmacological agents to improve mitochondrial quality and maintains mitochondrial functions under ischemia conditions or to develop multitarget drugs and multidrug therapies with mitochondria protection action via the NAMPT-NAD+ biosynthesis pathway [4, 12, 18]. Traditional herbal medicine and natural products commonly possess various pharmacological activities, such as antioxidation, anti-inflammation, antiapoptosis, mitochondrial function improvement, and neurofunctional regulation [29, 30]. Therefore, it is a promising strategy to look for neuroprotective natural agents with mitochondria protection actions mediated by NAMPT-NAD+ pathway, which has great importance for the development of novel drugs for the treatment of ischemic stroke.

Currently, notoginseng leaf triterpenes (PNGL), as total saponins of *Panax* notoginseng stem and leaf, are isolated and purified from stems and leaves of *Panax notoginseng (Burk) F. H. Chen*, which is widely confirmed as the raw materials of medicinal resource, functional foods, and common foods. Our research team used PNGL to have developed a new fifth of Chinese medicines—“*Qiye Tongmai Capsules*.” And we monitored eleven batches of PNGL samples by the chemical fingerprinting assay [31]. Our previous study has shown that PNGL exerts potent neuroprotective effects and antiapoptotic properties via attenuation of neuronal apoptosis caused by ischemia [31]. But the neuroprotective mechanisms of PNGL are not fully elaborated. In addition, our team previously has found that Panax notoginseng saponins (PNS) and its monomeric saponin components could reduce mitochondrial damages [32–34]. PNGL possessed pharmacological effects are similar to that of PNS. But it is essential to further verify whether PNGL exerts mitochondria-protective effects against cerebral I/R injury. And the relevant mechanisms are unclear that how PNGL may alleviate mitochondrial dysfunction, improve energy metabolism and thus suppress cerebral ischemic damages, which needs to further explore *in vitro*.

Therefore, this present research was to further explore the effects and mechanisms of PNGL against ischemic injury, mitochondrial injury, and metabolic disorder of energy in the OGD/R model *in vitro*, and FK866 was adopted to explore the regulation actions of PNGL on the NAMPT pathway.

## 2. Methods

### 2.1. Cell Culture

The human neuroblastoma cell line SH-SY5Y was obtained from the Institute of Basic Medical Sciences at the Chinese Academy of Medical Sciences and maintained in Dulbecco's modified eagle's medium (DMEM, NO. 1313831, Gibco, U.S.) with 10% fetal bovine serum (FBS, 1693361, Gibco, U.S.), 100 U/mL penicillin and 100 mg/mL streptomycin in a normal incubator, as a complete medium (CM) containing 4.5 g/L D-glucose. Cells incubated at 37°C in 95% humidity and 5% CO_2_. Confluent cells subcultured by diluting 1 : 3 in a 10 cm dish, and culture medium replaced once every two days.

### 2.2. OGD/R Induction

**Figure 1:**
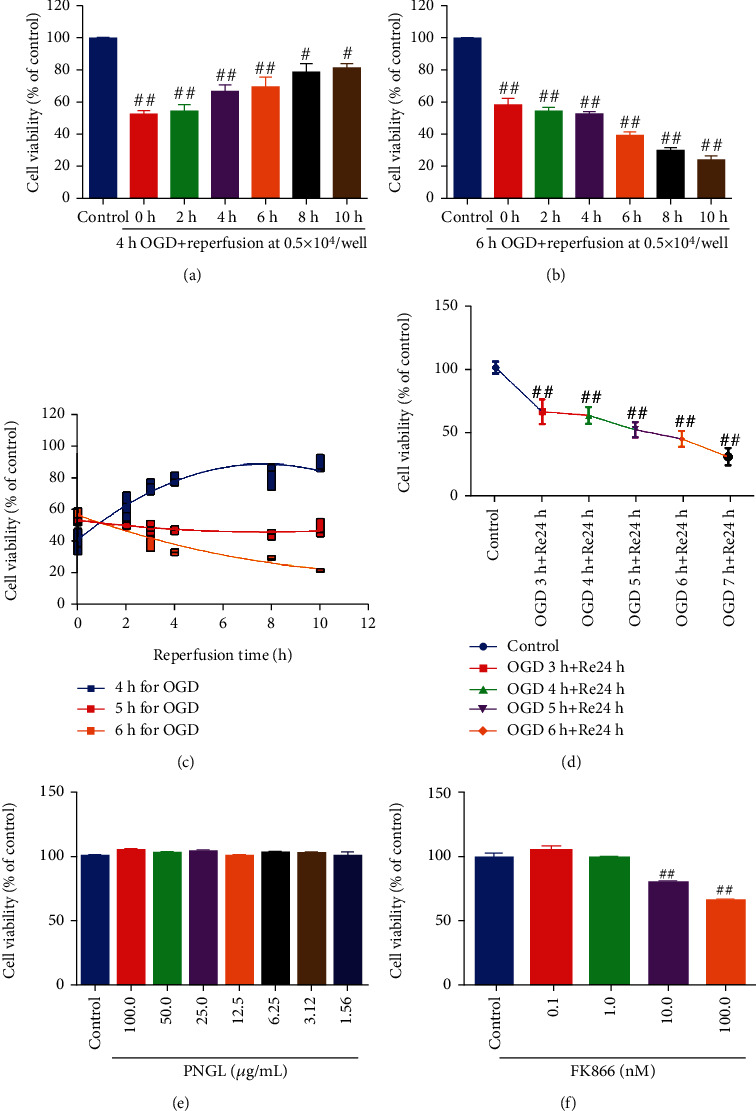
OGD/R model establishment, and effects of PNGL and FK866 on normal SH-SY5Y cell viability. The ischemia cell model condition was the OGD treatment for 4 h and followed 24 h reperfusion *in vitro*. (a–d) The effects of OGD followed reperfusion on SH-SY5Y cells for various times, which induce the OGD/R cell model. (e) The toxic effect of PNGL treatment on SH-SY5Y cells. (f) The toxic effect of FK866 treatment on SH-SY5Y cells. The copretreatment of PNGL and FK866 for 24 h, followed by OGD/R treatment. The cell viability was measured by using the MTT assay. The data presented as the mean ± standard error of the mean. ^#^*P* < 0.01, ^##^*P* < 0.01 versus the control group; ^∗^*P* < 0.05, ^∗∗^*P* < 0.01 versus the OGD/R model group; ^&^*P* < 0.05, ^&&^*P* < 0.01 versus the OGD+PNGL group.

To mimic ischemic-like conditions *in vitro*, SH-SY5Ycells were exposed to oxygen and glucose deprivation and reperfusion (OGD/R) as we described previously [35]. Briefly, cells washed with PBS, then replaced with glucose-free DMEM (11966025, Gibco, U.S.), and incubated in an anaerobic incubator (Type III, COY Laboratory, U.S.) under the conditions of 37°C, 95% N_2_, and 5% CO_2_ for different time (3 h, 4 h, 5 h, 6 h, 7 h, 8 h,). Cells were then reoxygenated and lasted for 24 h. The control cells were plated in DMEM containing glucose under normal oxygenation. Base on the current results (Figure 1), the conditions of OGD/R induction were the OGD for 4 h and reperfusion for 24 h.

### 2.3. Incubation and Treatment for Cells

The PNGL samples were obtained from by Jilin Academy of Chinese Medicine (LOT.2018-05-08). And we monitored eleven batches of PNGL samples by the chemical fingerprinting assay [31]. FK866, as a highly specific NAMPT inhibitor, could inhibit the NAMPT and NAD+ biosynthesis in mammals [23, 36, 37]. Our present experiment has suggested the potential mechanisms *in vivo*. To further explore the underlying mechanisms, FK866 (SECK-S2799, Selleck Chemicals, Beijing, China) was adopted.

The cell groups were showed as follows: the control group, the control group exposed to PNGL, the OGD/R group, the PNGL-pretreated groups exposed to OGD/R, the inhibitor Fk866-treated group exposed to OGD/R, and the coincubation group with the inhibitors and PNGL exposed to OGD/R. In the PNGL group, the cells were coincubated with different concentrations (1.56~100 *μ*g per mL) for another 24 h, then followed an insult of OGD/R. The inhibitors were added 1 h before PNGL treatment until the end of the experiment.

### 2.4. Cell Viability Assay

Cell viability was determined using a 3-(4,5-dimethylthiazol-2-yl)-2,5-diphenyltetrazolium bromide (MTT) assay [35, 38]. The absorbance was measured at 490 nm via a microplate reader (M1000, Tecan Infinite, Switzerland). Cell viability was expressed as a percentage with the control group as 100%.

### 2.5. Cell Apoptosis and Necrosis Assay

SH-SY5Y cell apoptosis and necrosis were detected by using an ANNEXIN V/Dead Cell.

Apoptosis kit (194785, Thermo Fisher Scientific, USA) and a Hoechst 33342/PI kit (CA1120, Solarbio, Beijing, China) according to the operation protocol [38, 39]. Briefly, after incubation and treatment, SH-SY5Y cells were harvested using 0.05% trypsin, centrifuged to remove the medium, washed twice with ice-cold PBS, and resuspended in 500 *μ*L of 1X binding buffer. The 5 × 10^4^ cells (30 *μ*L of cell suspension) were mixed and incubated with 5 *μ*L of Annexin V-FITc reagent at 37°C for 30 min under dark conditions, and followed by propidium iodide (PI, 5 *μ*L) staining for 5 min. Stained cells were measured via a FACSCanto flow cytometry (BD, Biosciences, USA). The percentage of cells in quadrant 2 (Q2) and 4 (Q4) were quantitatively analyzed, generating the total percentage of apoptotic cells (apoptotic index) both early and late apoptotic stage. All experiments were performed in triplicate.

Meanwhile, after incubation and treatment, cells were washed three times with FBS-free DMEM and then incubated with the 10 *μ*g/mL Hoechst 33342 staining solution for 20 min at room temperature in the dark, followed by PI staining for 5 min. Finally, after washed two times with DMEM, the cells were examined using a fluorescence microscope (EVOS M5000, Thermo Fisher Scientific, USA).

### 2.6. Measurement of Intracellular ROS

To measure intracellular ROS production, the 2′, 7′,-dichlorofluorescein diacetate (DCFH-DA) assay was adopted in vitro according to the manufacturer instructions (20181219, Solarbio, Beijing, China). In the presence of ROS, DCFH reacts with ROS to form the fluorescent product DCF. After incubation and treatment, cells were washed three times with DMEM and then incubated with a DCFH-DA staining solution (10 *μ*mol/L) 37°C for 30 min in the dark. A fluorescence microscope (EVOS M5000, Thermo Fisher Scientific, USA) was used to examine fluorescence values.

### 2.7. Determination of MMP

Mitochondrial membrane potential (MMP) was assessed as described previously [38, 39] using a JC-1 MMP assay kit (C2006, Beyotime, Shanghai, China) according to the operating manual. Briefly, cells were washed twice with FBS-free DMEM and then incubated with JC-1 (200 *μ*M) for 20 min at 37°C, followed by washing with DMEM to remove excess JC-1. MMP was calculated by the ratio of red to green fluorescence. The fluorescence images of JC-1 in the SH-SY5Y cells were acquired by a fluorescence microscopy (EVOS M5000, Thermo Fisher Scientific, USA).

### 2.8. Assessment of Mitochondrial Viability

After incubation and treatments, the mitochondrial viability was evaluated as described previously [40]. The MitoTracker Red CMXRos probe solution was prepared according to the manufacturer instructions. Cells were washed twice with DMEM and then incubated with a MitoTracker Red CMXRos probe (50 nM) for 45 min at 37°C, followed by washing with DMEM. The fluorescence images of mitochondrial viability in the SH-SY5Y cells were acquired by a fluorescence microscopy (EVOS M5000, Thermo Fisher Scientific, USA).

### 2.9. ELISA Assay

After incubation and treatment, SH-SY5Y cells were collected and sonicated by ultrasound homogenization in ice-cold lysis buffer. The supernatants from the ischemic brain tissues and the pretreatment cells were prepared by centrifugation (14,000 × g, 20 min). The ATP, ATPase, NAD, and NADH levels were detected by using ELISA kit (Hiton., Beijing, China). All experimental steps were performed according to the kit operation instructions. In addition, the protein concentrations of the collected supernatants *in vitro* were determined via the BCA protein assay kit (CWBIO, Beijing, China). OD values were measured by a microplate reader.

### 2.10. Protein Extraction

After SH-SY5Y cells were harvested and lysed in precooled RIPA buffer (CWBIO, Shanghai, China) containing with proteases and phosphatase inhibitor cocktail (1/100). After incubation on ice for 20 min, the tissue and cell lysates were then centrifuged at 14000 × g for 15 min at 4°C. The supernatant samples were collected. Then, the protein concentrations in the supernatant were determined by the BCA protein assay kit according to the protocol (CWBIO, Shanghai, China).

### 2.11. Western Blotting

Western blotting was performed as previously reported [38, 41]. Based on the manufacturer instructions, protein samples were loaded onto the SDS-PAGE gel, separated electrophoretically, and transferred onto NC membranes (Millipore, Bedford, MA, USA). After blocking the nonspecific binding sites for 2 h for 2 h at room temperature, the membranes were individually incubated overnight at 4°C with the related antibodies: NAMPT (ab236873, 1 : 1000), SIRT1 (ab189494, 1 : 1000), SIRT2 (ab211033, 1 : 2000), SIRT3 (cst5490, 1 : 1000), FOXO3A (ab109629, 1 : 3000), p-FOXO3A (ab52857, 1 : 1000), MnSOD (ab137037, 1 : 5000), PGC-1*α* (ab188102, 1 : 5000), and *β*-actin (EXP0041F, 1 : 3000). Then, the membrane was incubated at room temperature for 2 h with horseradish peroxidase-conjugated antibodies at a 1 : 2000 dilution. To eliminate variations in protein expression, three independent experiments were conducted.

### 2.12. Statistical Analysis

Data are presented as the mean values ± standard error of the mean (SEM). All analyses were performed by using the GraphPad Prism 8.0 statistical software (GraphPad Software, Inc., La Jolla, San Diego, CA, USA). Two-way analysis of variance (ANOVA) was used with drug and treatment as independent factors. Group differences after significant ANOVAs were measured by post hoc Bonferroni test, and *P* < 0.05 was considered statistically significant.

## 3. Results

### 3.1. OGD/R Treatment Induces an Ischemia and Hypoxia Cell Model *In Vitro*

Our previous research proved the neuroprotective effects of PNGL against cerebral I/R injury [42]. And it was preliminarily found that PNGL-induced mitochondrial protection *in vivo*. To further investigate the neuronal actions and mechanisms of PNGL against mitochondrial oxidative injury, an ischemia and hypoxia neuronal cell model was built to mimic the pathological changes of stroke *in vitro.* SH-SY5Y cells were exposed to OGD/R induction. MTT assay was employed to assess the cell viability.

The results showed that as cells were exposed to the OGD for 3-7 h periods then followed by 24 h of reperfusion, and cell viability has a time-dependent reduction, which reached 55% following 5 h of OGD (Figure 1(a), *P* < 0.01 versus control group); after exposure of OGD for 4 h, the cell viability was partially improved along with the reperfusion time (Figure 1(b) and 1(c), *P* < 0.01). However, with OGD for 6 h, the cell viability partially decreased and aggravated (Figure 1(b)–1(d), *P* < 0.01), indicating that the 5 h of OGD treatment may be the critical point for human neurons to resist ischemic injury [28, 43]. Hence, the 4 h OGD treatment was used as the conditions of subsequent experiments.

Moreover, FK866, as a highly specific NAMPT inhibitor, was used to block the NAMPT enzymatic function and inhibit the NAD synthesis neurons, which is to verify the protective effects and mechanisms of PNGL (Figure 1(e) and 1(f)). The results revealed that treatment with PNGL did not significantly inhibit cell viability at concentrations of 1.56 to 100.0 *μ*g/mL in SH-SY5Y cells (Figure 1(f)); FK866 (0.1~1 nm) showed no significant inhibition on neurons, but at the 10-100 nm, the cell viability obviously decreased (Figure 1(f), *P* < 0.01), which was in line with the reported concentration of 1.83 nM in SY5Y cells [20, 42, 44, 45]. Therefore, SH-SY5Y cells were pretreated with FK866 at concentrations of up to 1.0 nm for 1 h, and then followed by coincubation with PNGL for 24 h in the subsequent experiments.

### 3.2. PNGL Improves Cell Viability and Inhibits Apoptosis/Necrosis, Partly Reversed by FK866 *In Vitro*

**Figure 2:**
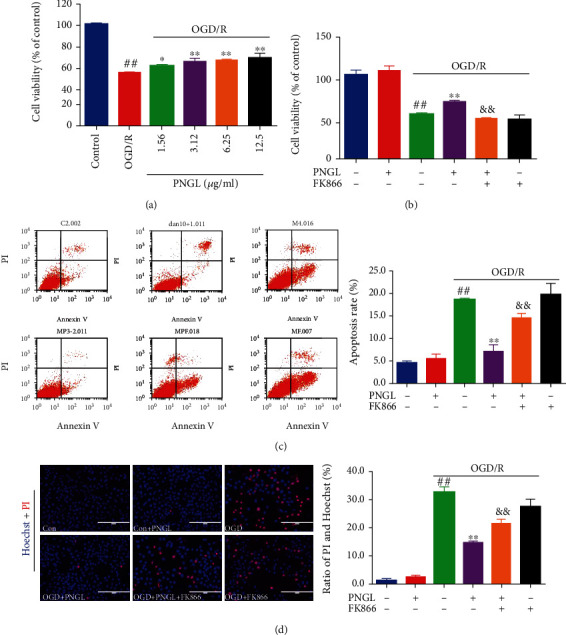
Effects of PNGL on cell viability and apoptosis in OGD-induced SH-SY5Y cells. PNGL improved the cell viability and inhibited the apoptosis rate in OGD-induced SH-SY5Y cells, which was partly reversed by the inhibitor FK866. (a) The pretreatment with PNGL (1.56~12.5 *μ*g/mL) for 24 h, followed by OGD/R treatment. (b) The copretreatment of PNGL and FK866 for 24 h, followed by OGD/R treatment. The cell viability was measured by using the MTT assay. (c) The annexin V/propidium iodide (PI) staining in OGD/R-induced SH-SY5Y cells, measured by a flow cytometer. (d) The hoechst33324/PI staining in OGD/R-induced SH-SY5Y cells, measured by a fluorescence microscope. The statistical analysis, analyzed by using the Image J 2.44 software. The data presented as the mean ± SEM (*n* = 3). ^#^*P* < 0.05, ^##^*P* < 0.01 versus the control group; ^∗^*P* < 0.05, ^∗∗^*P* < 0.01 versus the OGD/R model group; ^&^*P* < 0.05, ^&&^*P* < 0.01 versus the OGD/R+PNGL group. Scale bar, 200 *μ*m.

SH-SY5Y cells were simultaneously stained with AV−FITC/PI and Hoechst33324/PI, followed by the flow cytometry and fluorescence microscope analysis. It demonstrated that OGD-induced SH-SY5Y cell viability significantly decreased compared with the control group (Figure 2(a), *P* < 0.01); however, at concentrations of 1.56 to 12.50 *μ*g/mL, PNGL dose-dependently increased SH-SY5Y cell viability following OGD injury (Figure 2(a), *P* < 0.01), and, thus the concentration of 6.25 *μ*g/mL was selected to conduct the investigation in vitro.

Under normal conditions, a low level of neuronal apoptosis and necrosis levels were noted (Figures 2(c) and 2(d)). After the OGD/R inductions, the percentage of apoptotic and necrosis cells increased (Figures 2(c) and 2(d), *P* < 0.01), accompanied by the cell viability decreases (Figure 2(b), *P* < 0.01). In contrast, pretreatment with PNGL suppressed the apoptosis rates (Figure 2(d), *P* < 0.01) and improved cell viability (Figure 2(b), *P* < 0.01), respectively, but these effects of PNGL against neuronal injury were partly abolished by the further FK866 incubation (Figures 2(b)–2(d), *P* < 0.01). Besides, there were no significant differences in cell viability and apoptosis rates between control cells and PNGL-treated cells, which indicated that these concentrations of PNGL were nontoxic under normal conditions.

Hence, these results indicate that PNGL decreases the OGD-induced ischemia injury on SH-SY5Y cells *in vitro*, and the neuroprotective effects may be associated with the NAMPT.

### 3.3. PNGL Raises MMP and Reduced ROS Levels, Partly Reversed by FK866 *In Vitro*

Intracellular ROS production, stimulation, and reduction of MMP following OGD/R were thought to be important markers for the OGD/R-induced neurotoxicity and apoptosis. To evaluate the mitochondrial injury, we quantified the mitochondria membrane potential and intracellular ROS levels via JC-1 and DCFH-DA kits.

**Figure 3:**
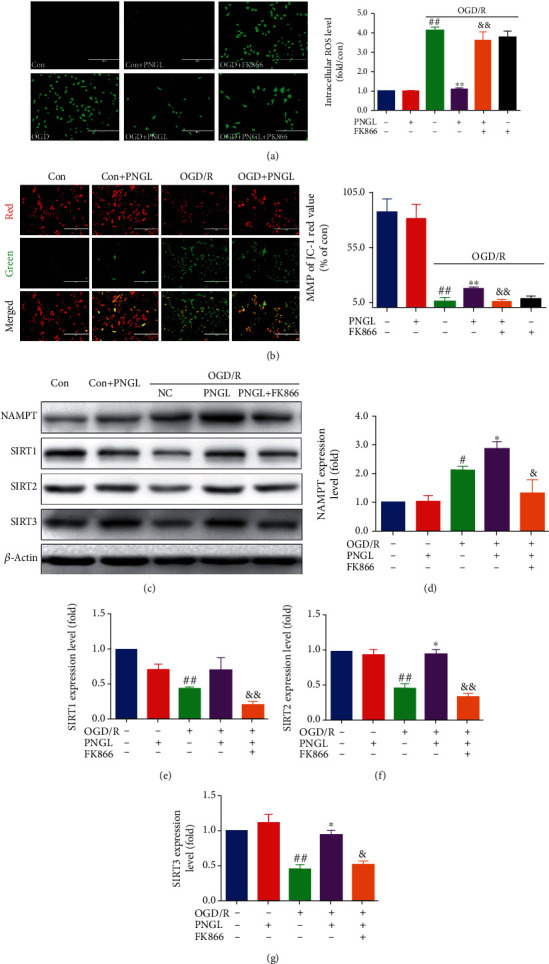
Effects of PNGL on the MMP, ROS levels, and the Nampt-SIRT1/2/3 pathway in OGD-induced SH-SY5Y cells. PNGL raised MMP, reduced the ROS levels, and upregulated the Nampt and SIRT1/2/3 expression in OGD/R-induced SH-SY5Y cells, partly reversed by the inhibitor FK866. (a) The ROS fluorescence images of PNGL in OGD/R-induced SH-SY5Y cells, measured by a fluorescence microscope. (b) The JC-1 fluorescence images of PNGL on MMP, measured with the JC-1 assay by a fluorescence microscope. The statistical data of fluorescence value, analyzed using the Image J 2.44 software; scale bar, 200 *μ*m. (c) The protein bands were examined by western blot analysis. (d–g) The relative expression levels were quantified and analyzed by using Gel-Pro analyzer software. The data presented as the mean ± SEM. ^#^*P* < 0.05, ^##^*P* < 0.01 versus the control group; ^∗^*P* < 0.05, ^∗∗^*P* < 0.01 versus the OGD/R model group; ^&^*P* < 0.05, ^&&^*P* < 0.01 versus the OGD/R+PNGL group.

Our data showed that the OGD/R group, compared to the control group, markedly increased intracellular ROS production (Figure 3(a), *P* < 0.01) and decreased the MMP levels (Figure 3(b), *P* < 0.01). Conversely, cotreatment with PNGL reduced intracellular ROS production (Figure 3(a), *P* < 0.01) and improved the MMP and the red value (Figure 3(b), *P* < 0.01). Treatment with PNGL alone shows no significant effect on ROS and MMP levels. Furthermore, the coincubation of FK866 partly reversed the ROS decreases and the MMP improvement (Figures 3(a) and 3(b), *P* < 0.01, *P* < 0.01).

These results suggest that PNGL has a vital role in the inhibition of mitochondrial injury, preservation of redox balance, and improvement of neuronal survival, which may be mediated by the target NAMPT.

### 3.4. PNGL Upregulates the NAMPT-SIRT1/2/3 Pathway, Partly Reversed by FK866 *In Vitro*

NAMPT, as a rate-limiting enzyme of NAD biosynthesis in the salvage pathway, positively regulates the activity of sirtuins and exerts protective effects in many cell types [46, 47]. To further explore the relationship between the PNGL and the NAMPT-NAD-mediated SIRT1/2/3 pathways *in vitro*, we also used FK866 to investigate *in vitro*, which are involved in neuronal energy metabolism and mitochondrial functions following neuronal oxidative stress.

As shown in Figure 3(c), as compared with the control group, the NAMPT expression level increased in the OGD/R group (Figure 3(d), *P* < 0.01). Treatments with PNGL increased the NAMPT expression, compared with the model group (Figure 3(d), *P* < 0.01). Interestingly, the SIRT1/2/3 expression was inhibited by OGD/R (Figures 3(e)–3(g), *P* < 0.01); conversely, PNGL significantly upregulated the SIRT1/2/3 expression (Figures 3(e)–3(g), *P* < 0.05). However, the regulation of the NAMPT-SIRT1/2/3 pathways was partly reversed by the coincubation and PNGL and FK866 (Figures 3(e)–3(g), *P* < 0.05, *P* < 0.01, *P* < 0.01, *P* < 0.05). Taken together, these results suggest that PNGL may exert protective effects against I/R injury via regulation of the NAMPT-SIRT1/2/3 pathways.

### 3.5. PNGL Improved Mitochondria and Energy Metabolism, Partly Reversed by FK866 *In Vitro*

**Figure 4:**
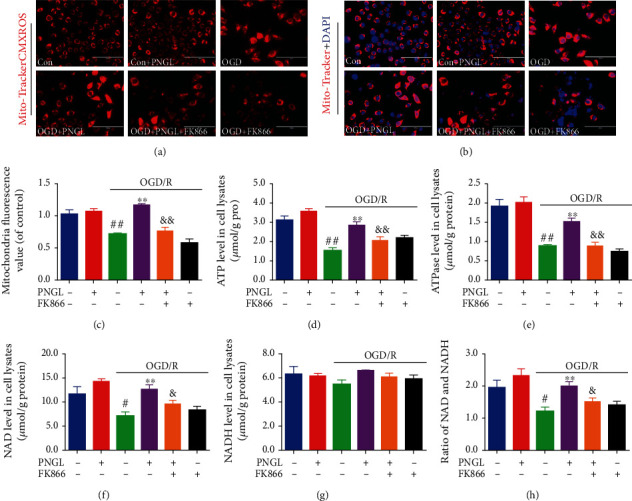
Effects of PNGL on mitochondria and energy metabolism in OGD-induced SH-SY5Y cells. PNGL improved mitochondria and energy metabolism, partly reversed by FK866, which was partly reversed by the inhibitor FK866. (a, b) The representative images of mitochondria, stained by the Mito-Tracker-CMXRos, were measured by a fluorescence microscope. (c) The statistical data of mitochondria fluorescence value was analyzed by using the ImageJ 2.44 software. (d, e) The ATP and ATPase levels in OGD/R-induced SH-SY5Y cells were detected by ELISA assay kits. (f, g) The NAD and NADH levels were detected by the ELISA assay kits. (h) The ratio of NAD/NADH levels in cells. The data presented as the mean ± SEM. ^#^*P* < 0.05, ^##^*P* < 0.01 versus the control group; ^∗^*P* < 0.05, ^∗∗^*P* < 0.01 versus the OGD/R model group; ^&^*P* < 0.05, ^&&^*P* < 0.01 versus the OGD/R+PNGL group. Scale bar, 100 *μ*m.

Mitochondria and mitochondrial metabolism play a vital role in the ischemia and hypoxia process; inhibition of mitochondrial viability leads to energy metabolism dysfunction and a cascade of mitochondria-dependent apoptosis [4, 48]. Hence, we assessed the mitochondrial structure and energetic metabolism *in vitro* via the FK866 and Mito-Tracker Red CMXRos assay (Figure 4).

In this study, OGD/R significantly decreased the mitochondrial viabilities, compared with the control group (Figures 4(a)–4(c), *P* < 0.01). Treatment with PNGL improved mitochondrial viability compared with the model groups (Figures 4(a)–4(c), *P* < 0.01), which was partly reversed by FK866. The ATP and ATPase levels decreased in OGD/R-induced SH-SY5Y cells (Figures 4(d) and 4(e), *P* < 0.01), but PNGL incubation increased the levels of ATP and ATPase (Figures 4(d) and 4(e), *P* < 0.01). In addition, the coincudation of FK866 and PNGL abolished the alteration (Figures 4(d) and 4(e), *P* < 0.01). These results suggest that PNGL has a crucial role in maintaining mitochondrial functions, which may be mediated by the NAMPT.

Next, we assessed the NAD+ and NADH levels mediated by the NAMPT in the OGD/R-induced SH-SY5Y cells. OGD/R induction significantly decreased intracellular NAD+ and NADH levels, and yet PNGL alleviated the energy metabolic disorder caused by OGD (Figures 4(f)–4(h), *P* < 0.01). And the FK866 partly blocked the improvement (Figures 4(f)–4(h), *P* < 0.05).

Taken together, all of these results suggest that PNGL effectively inhibits oxidative stress injury, alleviates energy metabolism dysfunction, improves mitochondrial function, and thus reduces the neuronal loss and apoptosis *in vitro*, which may be closely associated with the NAMPT and the SIRT1/2.

### 3.6. PNGL Improves the Downstream Foxo3a-MnSOD/PGC-1*α* Pathway, Partly Reversed by FK866 *In Vitro*

At last, we further determined the PGC-1*α*, MnSOD, and phosphorylated FOXO expression levels, as the critical downstreams of the NAMPT-NAD-SIRT1/2/3, which regulates transcriptional activity of the targeted antioxidant and cell cycle genes, including the UCP2, PECPK, PGC-1*α*, MnSOD, and catalase genes. The downstreams exert crucial homeostatic effects via inhibition of mitochondrial oxidative injury and improvement of mitochondrial energy recovery.

**Figure 5:**
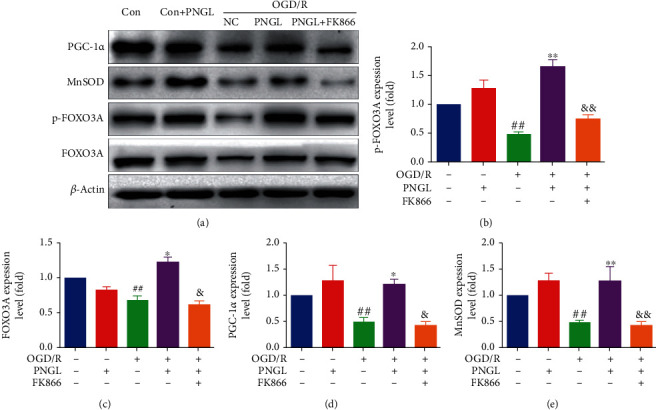
Effects of PNGL on the downstream SIRT1/2/3-Foxo3a-MnSOD/PGC-1*α* signaling pathway in the OGD/R-induced SH-SY5Y cells. PNGL regulated the MnSOD, PGC-1*α*, Foxo3a, and p-Foxo3a expression levels, which was partly reversed by the inhibitor FK866 *in vitro*. (a) The protein bands of the MnSOD, PGC-1*α*, Foxo3a, and p-Foxo3a were examined by western blot analysis in the OGD/R-induced SH-SY5Y cells. (b–e) The expression levels of the proteins were quantified and analyzed using the Gel-Pro analyzer software. The data presented as the mean ± SEM. ^#^*P* < 0.05, ^##^*P* < 0.01 versus the control group; ^∗^*P* < 0.05, ^∗∗^*P* < 0.01 versus the OGD/R model group; ^&^*P* < 0.05, ^&&^*P* < 0.01 versus the OGD/R+PNGL group.

As revealed in Figure 5, the OGD/R treatment decreased the phosphorylated Foxo3a expression levels (Figures 5(a)–5(c), *P* < 0.01) and downregulated the expression of the MnSOD and PGC-1*α* (Figures 5(d) and 5(e), *P* < 0.01). By contrast, treatment of PNGL effectively increased the expression of phosphorylated Foxo3a (Figure 5(b), *P* < 0.01), and accompanied by the upregulation of Foxo3a phosphorylation, the PGC-1*α* and MnSOD expression levels were also improved by PNGL (Figures 5(d) and 5(e), *P* < 0.05, *P* < 0.01). However, the incubation of FK866 suppressed the changes induced by PNGL.

## 4. Discussion

Ischemic stroke remains one of the leading causes of death worldwide, which is mainly caused by cerebral ischemia and reperfusion injury [6, 49]. Currently, scholars have done lots of researches on how to prevent and treat cerebral ischemia and reperfusion injury [6, 7]. Compared with the actual clinical application need, it is still seriously insufficient. Therefore, efficient drug treatments for ischemic stroke are urgently needed. In our previous works, PNGL exerted the neuroprotective effects against cerebral I/R injury via apoptosis suppression and mitochondrial protection [29, 31]. But the neuroprotective mechanisms of PNGL are not completely elaborated. In the present paper, the protection mechanisms of PNGL against I/R injury were further investigated. The study demonstrates that PNGL significantly improves cell viability (Figures 1 and 2), decreases the ROS levels, raised MMP (Figure 3), and inhibits apoptosis and necrosis (Figure 2). The *in vitro* results further suggest that PNGL is a promising agent for preventing and treating ischemic stroke.

Knowingly, a critical reduction of regional cerebral blood flow or severe oxygen and glucose deprivation leads to mitochondrial dysfunction within minutes after ischemia [11, 12], which could trigger mitochondrial quality control [9], including ROS scavenging, mitochondrial dynamics, and mitophagy [11, 12, 17]. Meanwhile, the related study demonstrates that depletion of ATP production is one of the major initiators, which triggers ischemic cascades, such as membrane ion pump failure, efflux of cellular potassium, influx of sodium, and membrane depolarization. Maintaining the mitochondrial function is critical in promoting neuron survival and neurological improvement [11, 12, 17]. Our research results showed that treatment with PNGL remarkably alleviated mitochondrial structure injury caused by OGD/R (Figure 4), increased ATP and ATPase levels (Figures 4(a)–4(e)), increased the mitochondrial viability (Figures 4(a)–4(e)), indicating that the neuroprotection of PNGL might be tightly associated with inhibiting mitochondrial injury and improving metabolism of energy.

**Figure 6:**
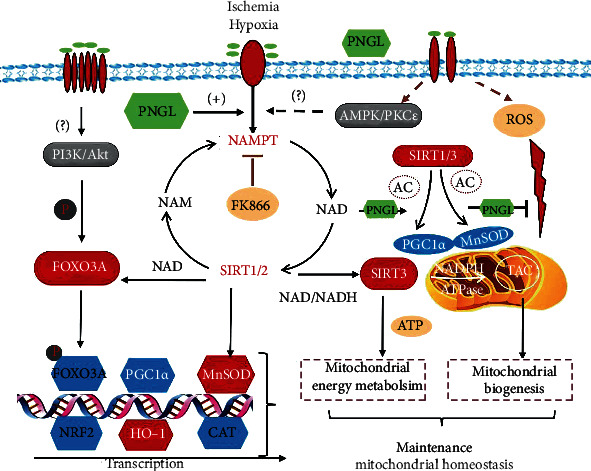
Effects and mechanisms of PNGL against cerebral ischemia injury via activating the NAMPT-NAD+ biosynthesis, regulating the key downstream SIRT1/2/3-Foxo3a-MnSOD/PGC-1*α* signaling pathways, and thus inhibiting mitochondrial oxidative damages, improving energy metabolism, and alleviating neural apoptosis caused by ischemia and hypoxia (tricarboxylic acid cycle, TAC).

NAD+, as a coenzyme, plays a vital role in energy balance and cellular redox reactions in ischemic stroke [50–52]. In the cytosol, NAD is translated to NADH during glycolysis, and the tricarboxylic acid cycle (TAC) enzymes reduce the NAD+ molecules [9, 52]. Under the hypoxia-ischemia conditions, NADH gets oxidized in the cytoplasm via the reduction of pyruvate to lactate (Figure 6), which leads to mitochondria dysfunction and NAD decrease. And then ATP synthesis was inhibited [11, 18, 23]. Thus, the absence of the mitochondrial NAD+ pool causes oxidative damages and excessive ROS production [28, 50, 52], which aggravates mitochondria impairment, including the function of mitochondria structure, depletion of ATP production, and depolarization of MMP [12, 13]. In our study, the results showed after OGD/R induction, the NAD+ and the ratio of NAD+/NADH level significantly decreased (Figure 4), the oxidative injuries increased (Figure 3) *in vitro*, and MMP depolarized, which was consistent with the previous reports. While PNGL pretreated OGD/R-induced cells, it reversed these alterations of NAD+, ROS, MMP, and ATP caused by I/R. All of these indicated that PNGL might exert mitochondria protective effects via the maintenance of the mitochondrial NAD+ pool and inhibition of oxidative injury *in vitro*.

NAMPT is the rate-limiting enzyme in the NAD biosynthetic pathway [18, 53, 54]. As shown in Figure 6, intracellular NAMPT converts nicotinamide into NAD as the rate-limiting enzyme for mammalian NAD+biosynthesis [55–58]. The *in vitro* experiments demonstrated that the intracellular NAMPT level was induced by ischemia and OGD along with the NAD+ decrease (Figure 3(c)), which was in accordance with the related previous researches. In contrast, PNGL treatment markedly improved the intracellular level in OGD-induced cells (Figure 3(c)). All of these results indicate that PNGL may regulate the NAMPT pathway against the mitochondria dysfunction and cerebral I/R injury.

FK866, a potent inhibitor of NAMPT, significantly inhibits NAD biosynthesis [36, 59], which verifies that the NAMPT-mediated systemic NAD biosynthesis plays a critical role in the regulation of mitochondrial functionality [8, 23]. Hence, FK866 was adopted to demonstrate whether PNGL might regulate the NAMPT-NAD+ pathway *in vitro*. Our results showed that FK866 incubation partly reversed the improvement of NAD induced by PNGL (Figures 4(f)–4(h)), aggravated the mitochondrial injury, impaired the mitochondrial homeostasis (Figures 4(a)–4(c)), and further blocked the metabolic energy (Figures 4(d) and 4(e)). All these indicate that PNGL exerts mitochondrial protective effects via the NAMPT-NAD+ pathway. Moreover, accompanied by the NAD pool impairment and the deteriorated dysfunction of mitochondria (Figures 4(a)–4(c)), FK866 partly inhibited the antioxidant capability (Figure 3), the MMP improvement, and the neural cell viability (Figure 2), which indicated that the mechanisms might be closely associated with the NAMPT-mediated systemic NAD biosynthesis.

Sirtuins are an evolutionarily conserved family of NAD+-dependent lysine deacetylases and ADP ribosylases in mammals [8, 10, 52]. However, not all sirtuin members are strictly lysine deacetylates. SIRT1, SIRT2, and SIRT3 have the highest deacetylase activities and can exert great neuroprotective effects in cerebral ischemia [60–62]. With an NAD+-dependent deacetylase, SIRT1, SIRT2, and SIRT3 specifically promote the transcription of a set of genes related to cell survival [63–65], mitochondrial function [66], energy metabolism [26, 66, 67], oxidizing reaction [65, 66, 68], and inflammation [69, 70]. SIRT1/2/3 deficiency or knockdown attenuated the neuroprotection of NAD+ [52, 62]. The further research results suggested OGD/R treatment reduced the expression of SIRT1/2/3 *in vitro* (Figures 4(e)–4(g)), which was partly reversed by the FK866 *in vitro* (Figures 3(e)–3(g)). These findings indicate that SIRT1/2/3 may be involved in the regulation of PNGL.

SIRT1 and SIRT2 are key regulators of cellular antioxidative and antiapoptotic responses [26, 71, 72], and similar to SIRT1/2, NRF2 plays a crucial role in promoting mitochondrial biogenesis and regulating mitochondrial function with a relatively independent manner [43, 58, 73]. Under cellular stresses, SIRT1/2 can deacetylate FOXO1 and FOXO3A [68, 72, 74], and the transcriptional activity mediated by NRF2 is improved, which in turn, induces the increase of antioxidant genes expression, decrease of ROS production, and upregulation of mitochondrial superoxide dismutase (MnSOD) expression [58, 68]. In this study, OGD/R induction led to the SIRT1/2 downregulation, decrease of FOXO3a nuclear localization and phosphorylated levels (Figures 5(e)–5(g)), increase of the ROS production, downregulation of antioxidant proteins and factors (SOD, CAT, MnSOD), and mitochondria injury (Figures 5(e)–5(g)). However, PNGL treatment upregulated the FOXO3a nuclear localization and phosphorylated levels (Figures 3 and 5) and improved the expression of antioxidant proteins *in vitro* (Figure 5), which was partly abolished by the FK866 (Figures 3 and 5). These results suggest that the effects of PNGL against I/R injury may be via regulating the NAMPT-NAD+ and SIRT1/2-FOXO3a-MnSOD pathways.

In addition, SIRT1 is not the only mediator of NAMPT to maintain mitochondrial NAD+ pool [75]. Sirt3 is the primary mitochondria-targeted deacetylase, predominantly expressed in highly metabolic tissues, and binds to and deacetylate several metabolic and respiratory enzymes that regulate mROS generation and mitochondrial functions [60, 62, 69]. Mitochondrial Sirt3 induces forkhead box O3 (FoxO3a) translocation to the nucleus and augments FoxO3a-dependent antioxidant defense mechanisms [68, 72, 76] through upregulation of PGC-1*α* [62, 65, 75] and SOD2 [66, 75], which is similar to the regulatory role of SIRT1. PGC-1*α* and SOD2 stimulate mitochondrial biogenesis and electron transport activity [14, 62, 65]. And they suppress ROS production and protect cells from mROS-induced oxidative damages. Thus, the SIRT1/3-mediated PGC-1*α* and SOD2 may be the critical proteins. Our data indicated that treatment with PNGL increased the PGC-1*α* and SOD2 levels in the OGD/R-induced cells (Figure 5), resulting in the inhibition of mitochondrial oxidizing injury, upregulation of the mitochondria TAC enzymes, improvement of energy metabolism, and maintenance of mitochondrial homeostasis. All of these results were further confirmed by FK866.

Overall, our results (Figure 6) suggest that PNGL can possess neuroprotective effects against cerebral I/R injury, notedly exert antioxidative and mitochondria-protective effects, improve the energy metabolism, and thus inhibit neuronal apoptosis and necrosis. The underlying mechanisms may be tightly associated with the NAMPT-NAD+ biosynthesis pathway and its downstream SIRT1/2/3-Foxo3a-MnSOD/PGC-1*α* signaling pathways. However, the molecular mechanisms of the mitochondrial biogenesis have not been completely elaborated. Therefore, further investigations will be required to more deeply elucidate.

## 5. Conclusions

In the present study, we explored and verified the protection effects and mechanisms of PNGL in OGD/R-induced SH-SY5Y cells. As elaborated in Figure 6, PNGL strikingly improves cell viability, significantly preserves redox balance, inhibits excessive ROS levels, alleviates mitochondrial injury, improves energy metabolism function (MMP, NAD, ATP, and ATPase levels), raises neuronal mitochondrial viability, reduces the neuronal necrosis and apoptosis, and thus notedly improves neuronal survival under ischemia and hypoxia conditions. In general, this study finds that the protective effects of PNGL are, at least partly, mediated through the NAMPT-NAD+ pathway and its key downstream SIRT1/2/3-Foxo3a-MnSOD/PGC-1*α* signaling pathways. PNGL, as a new drug candidate, has excellent application prospects for CIS.

## Data Availability

The data and drug samples used to support the findings of this study are available from the corresponding author upon request.

## Abbreviations

ATP: Adenosine triphosphate
CIS: Cerebral ischemic stroke
NAD: Nicotinamide adenine dinucleotide
ETC: Electron transport chain
FoxO3a: Forkhead box O3
I/R: Ischemia and reperfusion
MMP: Mitochondrial membrane potential
NO: Nitric oxide
MOJ: Mitochondrial oxidative injury
NAMPT: Nicotinamide phosphoribosyltransferase
OGD/R: Oxygen and glucose deprivation and reperfusion
PNGL: Notoginseng leaf friterpenes of *Panax* notoginseng stem and leaf
ROS: Reactive oxygen species
SOD: Superoxide dismutase
SIRT: Sirtuin
DMEM: Dulbecco's modified eagle's medium
MnSOD: Manganese superoxide dismutase
PGC-1*α*: PPAR*γ* coactivator-1*α*
TAC: Tricarboxylic acid cycle.
